# Expression of ATP/GTP Binding Protein 1 Has Prognostic Value for the Clinical Outcomes in Non-Small Cell Lung Carcinoma

**DOI:** 10.3390/jpm10040263

**Published:** 2020-12-02

**Authors:** Hee Jeong Kwak, Minchan Gil, Hee Sung Chae, Jaekwon Seok, Nagasundarapandian Soundrarajan, Subbroto Kumar Saha, Aram Kim, Kyoung Sik Park, Chankyu Park, Ssang-Goo Cho

**Affiliations:** 1 Department of Stem Cell and Regenerative Biotechnology, Incurable Disease Animal Model & Stem Cell Institute (IDASI), Konkuk University, 120 Neungdong-ro, Gwangjin-gu, Seoul 05029, Korea; h_jeong9581@naver.com (H.J.K.); minchangil@gmail.com (M.G.); gmltjdgk@konkuk.ac.kr (H.S.C.); tjrwornjs@naver.com (J.S.); sundarmeets@gmail.com (N.S.); subbroto@konkuk.ac.kr (S.K.S.); chankyu@konkuk.ac.kr (C.P.); 2 Department of Urology, Konkuk University Medical Center, Konkuk University School of Medicine, Seoul 05029, Korea; arkim@kuh.ac.kr; 3 Department of Surgery, Konkuk University Medical Center, Konkuk University School of Medicine, Seoul 05029, Korea; 20090117@kuh.ac.kr

**Keywords:** AGTPBP1, lung cancer, expression level, prognosis, patient survival

## Abstract

ATP/GTP binding protein 1 (*AGTPBP1*) encodes a crucial protein, cytosolic carboxypeptidase 1 (CCP1), which plays a role in modulating the polyglutamylation of tubulin and has been studied in degenerative diseases. However, the role of *AGTPBP1* in malignancy has not been completely studied yet. In this study, we examined the role of *AGTPBP1* in cancer progression, its association with patient survival, and related mechanisms in lung cancer, using the A549 cell line and lung cancer gene expression datasets. *AGTPBP1* knockdown increased the proliferation, migration, sphere formation, and drug resistance of A549 cells. Lung cancer datasets revealed significantly lower mRNA and protein expression levels of *AGTPBP1* in lung cancer tissues, as compared to those in normal tissues. Importantly, *AGTPBP1* expression positively correlated with patient survival. Analysis of co-expressed genes revealed that *AGTPBP1* expression positively correlated with immune infiltration in lung cancer. Our results conclusively suggested that *AGTPBP1* expression was correlated with cancer progression and immune infiltration in lung cancer.

## 1. Introduction

ATP/GTP binding protein 1 (AGTPBP1) encodes the protein, cytosolic carboxypeptidase 1 (CCP1), which mediates deglutamylation to maintain steady-state levels of polyglutamylated tubulin [1]. *AGTPBP1*, also known as nervous system nuclear protein induced by axotomy 1 (*Nna1*), was initially identified in nuclear processes associated with differentiating and regenerating neurons [2]. Furthermore, *AGTPBP1* expression has been detected at high levels in the motor neurons and testis of mice [2], and loss-of-function of CCP1 is associated with neurodegeneration and defective spermatogenesis in Purkinje cell degeneration (*pcd*) mice [3,4,5,6]. It has been revealed that CCP1 catalyzes the removal of glutamate residues from both the polyglutamyl side chanins of α- and β-tubulin and detyrosinated α-tubulin and induces the polymerization of α-tubulin [1,7]. These molecular functions can maintain steady-state levels of polyglutamylated microtubules in neurons [8], axoneme [9], mitotic spindles [10], ciliary dynein [11], and basal bodies [12]. Thus, the expression of *AGTPBP1* modulates the organization of microtubules and cellular dynamics and has direct effects on cell function and cilia wellness [13]. Since microtubules are essential components for cell division and migration, altered polyglutamylation of α- and β-tubulins is associated with tumorigenesis and drug resistance in patients with prostate cancer and neuroblastoma [14,15,16]. However, the role of *AGTPBP1* in human malignancy has not been comprehensively studied yet.

Lung cancer is one of top leading causes of cancer death in most countries and is classified into two main types, namely, small cell lung carcinoma (SCLC) and non-small cell lung carcinoma (NSCLC). Approximately 85% of patients with lung cancer suffer from NSCLC, of which lung adenocarcinoma (LUAD) and lung squamous cell carcinoma (LUSC) are the most prevalent subtypes [17]. According to a survey, lung cancer caused more deaths in 2017 than breast, prostate, colorectal, and brain cancers combined [18]. Among them, the five-year relative survival rate was 24% for NSCLC and 6% for SCLC [18]. In order to increase the survival rate for lung cancer, several differentially expressed genes, which are implicated as therapeutic targets and prognostic markers, have been investigated. In NSCLC, deregulated tubulin dynamics by the altered expression of class III β-tubulin results in poor patient survival [19]. Class III β-tubulin-silencing in NSCLC cells increased cell death at low concentration of two major microtubule-targeted chemotherapeutic drug [20]. Furthermore, the expression of Class V β-tubulin is negatively associated with cancer patient with taxane-based chemotherapy [21]. In normal lung tissue, the expression of *AGTPBP1* is relatively higher than other tissues [22]. CCP1, encoded by *AGTPBP1,* mediates the deglutamylation of tubulin, which could influence tubulin dynamics and the microtubule network in lung cancer [23]. Thus, investigation of the *AGTPBP1* functions is required for a better understanding in tubulin homeostasis in lung cancer.

In this study, we examined the effect of *AGTPBP1* on the proliferation, migration, and cancer stemness of lung cancer cells in vitro by silencing *AGTPBP1* with short-hairpin RNA (shRNA). The prognostic value of *AGTPBP1* and its associated pathways in lung cancer were investigated by analyzing the publicly accessible lung cancer datasets. Our results indicated that *AGTPBP1* expression in lung cancer tissues was lower than in normal counterparts and positively correlated with overall patient survival in lung cancer. *AGTPBP1* expression also correlated with immune infiltration in lung cancer. Therefore, our study revealed the role of *AGTPBP1* in lung cancer and its prognostic significance in patient survival.

## 2. Materials and Methods

### 2.1. Cell Line and Culture Condition

The human lung adenocarcinoma cell line A549 was obtained from Korean Cell Line Bank, Seoul, Korea and cultured in RPMI 1640 (Sigma-Aldrich, St. Louis, MO, USA) supplemented with 10% fetal bovine serum (Peak Serum, Wellington, CO, USA) and 1% penicillin/streptomycin (Gibco, Life Technologies, Grand Island, NY, USA). Cells were maintained at 37 °C in a humidified atmosphere of 5% CO_2_ with continuous monitoring for cell adherence and morphology using microscopy.

### 2.2. AGTPBP1 Knockdown Using Lentiviral Vector

Lentiviral plasmid for *AGTPBP1* knockdown (sh*AGTPBP1*) and lentiviral control plasmid (scramble) were purchased from Vector Builder (Chicago, IL, USA). Lentivirus were produced from the packaging cells transfected with lentiviral packaging (psPAX2) and envelop (pCMV-VSV-G) plasmids using Lipofectamine 3000 reagent (Invitrogen, Carlsbad, CA, USA) according to the manufacturer’s guidance. The sequences of shRNA targeting *AGTPBP1* were as follows: sense, 5′—aataattagactctggcattgctgt—3′; and antisense, 5′—ttattaatctgagaccgtaacgaca—3′. After 24 h of transfection, the culture medium was changed with fresh medium and incubated for 48–72 h at 37 °C in a humidified atmosphere of 5% CO_2_. The culture supernatant was collected and filtered using a 0.45 μm syringe filter to prepare lentiviral soup, which was further used for infection of the A549 cell line.

### 2.3. Isolation of Total RNA Extraction and Reverse Transcription Polymerase Chain Reaction (RT-PCR)

Total RNA was acquired using Labozol reagent (LaboPass, CMRZ001, Cosmogenetech, Seoul, Korea) according to the manufacturer’s instructions. The extracted RNA was quantified using NanoPhotometer (IMPLEN, München, Germany). cDNA was obtained from 2 μg of total RNA and oligo dT primer using M-MuLV reverse transcription kit (LaboPass, CMRT010) and subjected to PCR analysis using rTaq Plus 5X PCR Master Mix (Elpisbiotech, EBT-1319). PCR products were subjected to 1–2% agarose gel electrophoresis. Band densities were analyzed using the Image J version 1.52p (National Institutes of Health, Bethesda, MD, USA) and calculated as a relative expression after normalization against the housekeeping gene *GAPDH*. The PCR primer sequences are given in Supplementary Table S1.

### 2.4. Cell Proliferation and Viability Assay

For analysis of cell proliferation, *AGTPBP1*-silenced cells and scrambled control (5 × 10^4^ cells/well) were seeded into 12-well culture plates. The number of cells was counted every 24 h up to day 5 using a hemocytometer after trypan blue exclusion. For cell viability analysis, cells (2 × 10^3^ cells/well) were seeded into 96-well culture plates and cultured, and 10% (*v*/*v*) Cell Counting Kit-8 solution (CCK-8; Dojindo, CK04-05, Kumamoto, Japan) was added to the wells at the indicated time points. After 3 h incubation in the same culture condition, the absorbance of the wells was measured at 450 nm using Bio-RAD x-MarkTM microplate spectrophotometer (Bio-Rad Laboratories, Hercules, CA, USA).

### 2.5. Cell Migration Assay

To analyze the migratory ability of the cells, cells were cultured to reach 95% confluency into 6-well plate and treated with 10 μg/mL of mitomycin for 3 h. The cell layer was scratched with the narrow end of a 1000-μL pipette tip and cultures after complete removal of debris caused by scratch. The wound areas in the dishes were marked and photographed every 24 h. The filled area by the moved cells was estimated using TScratch (Version 1.0, Swiss Federal Institute of Technology, Zurich, Switzerland) to measured closure percentage (%).

### 2.6. Sphere-Forming Assay

For the sphere-forming assay, 6 × 10^4^ cells were seeded into non-coated 6-well plates containing serum-free DMEM/F12 medium supplemented with B27 supplement, 20 ng/mL epidermal growth factor (Sigma Aldrich, Saint Louis, MO, USA), 10 μg/mL insulin (Sigma Aldrich), and 1% bovine serum albumin (Sigma Aldrich) [24], and incubated at 37 °C in a humidified atmosphere of 5% CO_2_ for five days. Then, colonies were harvested and stained with crystal violet (Sigma Aldrich) in 15 mL conical tubes (SPL Lifesciences, Pochen, Korea). Photographs of the spheres were analyzed using Image J software to determine sphere size.

### 2.7. Drug Resistance Assay

For drug-resistance assays, 3 × 10^3^ cells were seeded into 96-well plates and incubated overnight at 37 °C in a humidified atmosphere of 5% CO_2_. Next, the cells were exposed to doxorubicin (0.1, 0.2, 0.5, 1, 5, and 10 μM) and cisplatin (5, 10, 20, 50, 100, and 150 μM) for another 36 h at 37 °C in a humidified atmosphere of 5% CO_2_. After 36 h of incubation, 10% (*v*/*v*) CCK-8 solution was added to the cells and incubated for ~3 h. Relative absorbance of the wells was determined at 450 nm using Bio-RAD x-MarkTM spectrophotometer (Bio-Rad).

### 2.8. Analysis of AGTPBP1 mRNA Expression Pattern in Lung Cancer

Distribution pattern of *AGTPBP1* expression in various normal tissues was obtained from the Human Protein Atlas (HPA) version 19.3 (KTH, UU, SciLifeLab, Solna, Sweden) (https://www.proteinatlas.org) [25]. Subsequently, relative mRNA expression pattern of *AGTPBP1* in lung cancer tissues and its normal cellular counterparts was determined using the Oncomine database version 4.5 (Thermo Fisher Scientific Inc., Ann Arbor, MI, USA) (https://www.oncomine.org/resource/main.html), a web-based database and data-mining platform for mRNA expression [26]. The mRNA expression patterns of lung cancer tissues and corresponding normal tissues were compared using Student’s *t*-test with a *p*-value threshold < 1 × 10^−4^. Query with *AGTPBP1* was carried out in default setting to obtain the expression pattern of *AGTPBP1* in The Cancer Genome Atlas (TCGA) dataset using the Gene Expression Profiling Interactive Analysis (GEPIA) (Beijing, China) (https://gepia.cancer-pku.cn/) [27] and the UALCAN databases (Preston, Lancashire, UK) (https://ualcan.path.uab.edu/index.html) [28]. Differences were considered statistically significant at *p*-value < 0.01 and fold change cutoff > 2.

### 2.9. Analysis of AGTPBP1 Protein Expression Pattern in Lung Cancer

The protein expression levels of *AGTPBP1* in lung cancer tissues and normal tissues were acquired from the UALCAN and HPA web servers. Protein expression level of *AGTPBP1* was systematically analyzed using default settings based on the characteristics of patients with LUAD, derived from the Clinical Proteomic Tumor Analysis Consortium (CPTAC). Differences with *p*-value < 0.05 were considered statistically significant. The protein expression level of *AGTPBP1* between lung cancer tissues and normal tissues were compared using the HPA dataset. The protein expression levels of *AGTPBP1* in lung cancer tissues and normal tissues were analyzed by immunohistochemical staining of normal pneumocytes of patient ID 1678 and lung cancer tissues of patient ID 447 using anti-AGTPBP1 antibody, HPA057208. Antibody staining scored the staining intensity and fraction of the stained cell, indicating brown staining via the antibody labeled with 3,3′-diaminobenzidine.

### 2.10. Analysis of AGTPBP1 Expression and Survival Pattern in Lung Cancer

The relationship between *AGTPBP1* expression and prognosis of patients with lung cancer was investigated using the Kaplan-Meier Plotter (KM-plotter) (Semmelweis University, Budapest, Hungary) (http://kmplot.com/analysis/) [29], PrognoScan (Kyushu Institute of Technology, Fukuoka, Japan) (http://dna00.bio.kyutech.ac.jp/PrognoScan/) [30], and R2: Genomics Analysis and Visualization Platform (Academic Medical Center, Amsterdam, The Netherlands) (http://hgserver1.amc.nl/) [31] web tools. The Cox *p*-value threshold was set <0.05 to determine statistical significance.

### 2.11. Analysis of AGTPBP1 Alteration Frequency in Lung Cancer

The alteration frequency and copy number alterations (CNAs) of *AGTPBP1* gene in lung cancer were estimated using cBioPortal database (Center for Molecular Oncology at MSK, New York, NY, USA) (https://www.cbioportal.org/) [32]. cBioPortal is an open-access resource for interactive exploration of multidimensional cancer genomics datasets, which currently provides access to data from 283 cancer studies. Query with *AGTPBP1* was carried out using 4744 samples from 17 combined studies of lung cancer and the alteration status of each was examined. Correlation between *AGTPBP1* expression and CNAs in LUAD and LUSC was examined using the TCGA PanCancer datasets. Furthermore, co-occurrence pattern between *AGTPBP1* and epidermal growth factor receptor (*EGFR*), ROS proto-oncogene 1 (*ROS1*), B-Raf proto-oncogene (*BRAF*), anaplastic lymphoma receptor tyrosine kinase (*ALK*), and KRAS proto-oncogene (*KRAS*) was investigated using the comparison/survival modules in cBioPortal web.

### 2.12. Analysis of Genes Co-Expressed with AGTPBP1 and Their Pathways

Genes co-expressed with *AGTPBP1* were explored in five different datasets of NSCLC using the R2: Genomics Analysis and Visualization Platform with adjustment of false discovery rate and *p*-value threshold < 0.01. The common co-expressed genes from different datasets were obtained using Venn diagrams. Next, the pathway and gene ontology shared by the co-expressed genes were evaluated using the Reactome analysis tool (Hinxton, Cambridge, UK) (https://reactome.org/) [33].

### 2.13. Analysis of Correlation between AGTPBP1 and Infiltration of Immune Cells

A comprehensive analysis of immune infiltration across diverse cancer types was performed using the Tumor IMmune Estimation Resource (TIMER) version 2.0 web tool (Liu Lab, Harvard university, Boston, MA, USA) (https://cistrome.shinyapps.io/timer/) [34]. The correlation between *AGTPBP1* expression and six tumor-infiltrating immune subsets (B cells, CD4^+^ T cells, CD8^+^ T cells, neutrophils, macrophages, and dendritic cells) was explored in LUAD using TIMER. The correlation between *AGTPBP1* expression and effector cell subsets was analyzed using the correlation modules in the TIMER web server. 

Additionally, to determine the relationship between human immune cell types and *AGTPBP1* expression, we utilized the Database of Immune Cell Expression, Expression quantitative trait loci (eQTLs) and Epigenomics (DICE) (La Jolla Institute for Immunology, San Diego, CA, USA) (https://dice-database.org/landing) [35]. DICE provides the opening data associated with human immune cell types and the eQTLs of unique genes. Cis-eQTLs for a total of 12,254 genes, which comprises 61% of all protein-coding genes expressed in immune cell types, have been identified using DICE. We used the keyword “AGTPBP1” in the Explore gene modules and the log scale was set as transcripts per million (TPM).

### 2.14. Statistical Analysis

All experiments were performed in triplicate. Mean values were determined with standard deviation. Statistical significance of differences was assessed using a two-tailed *t*-test in experiments and one-way analysis of variance (ANOVA) followed by Brown-Forsythe test in the cBioPortal database. Significance value is indicated on each graph: ns, non-significant, * *p* < 0.05; ** *p* < 0.01; *** *p* < 0.001.

## 3. Results

### 3.1. Cell Proliferation, Migration, Sphere Formation, and Drug Resistance in A549 Cells

**Figure 1:**
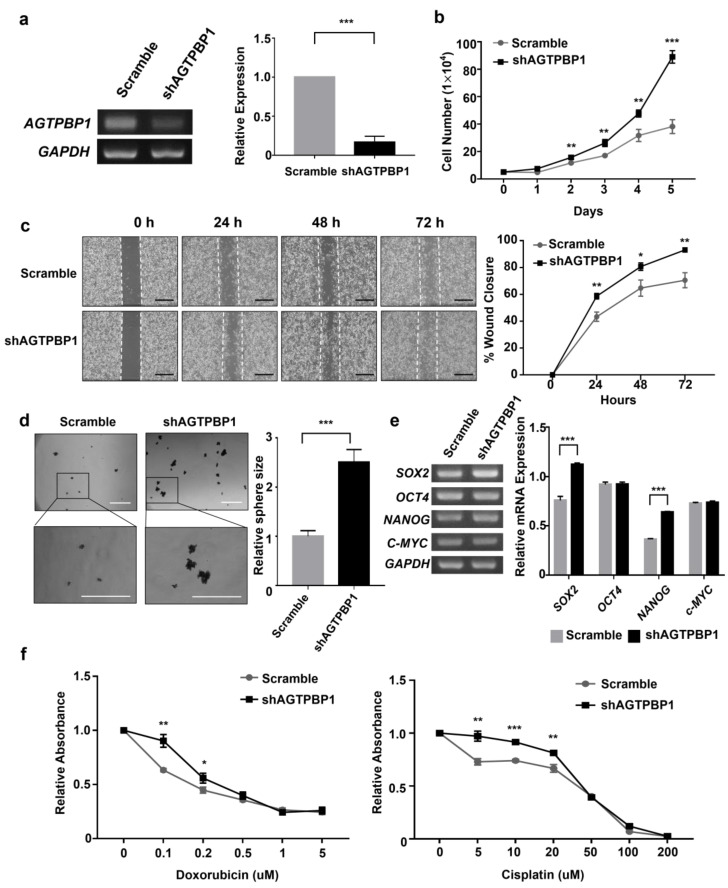
ATP/GTP binding protein 1 (*AGTPBP1*) silencing in the A549 lung cancer cell line. (**a**) Relative expression of *AGTPBP1* in sh*AGTPBP1*-transduced and control cells was analyzed using Reverse transcription polymerase chain reaction (RT-PCR). Glyceraldehyde 3-phosphate dehydrogenase *(GAPDH)* was used as the loading control. (**b**) The number of *AGTPBP1*-silenced cells and control cells was counted using a hemocytometer after trypan blue exclusion five days post transfection. (**c**) Cell migration analysis. The left panel represents wound closure of control and *AGTPBP1*-silenced cells. The right panel represents the percentage of each wound closure at 24, 48, and 72 h after initiation of wound closure. (**d**) Sphere forming assay was performed using 6 well non-coated culture plates over a period of five days. Sphere size was measured using Image J and is shown as the relative size of spheres formed by *AGTPBP1*-silenced cells, as compared to scramble cells. Scale bars correspond to 500 μm. (**e**) Expression of stemness markers was measured using RT-PCR. Expression level of genes were adjusted to the expression of internal control GAPDH and shown as bar graph. (**f**) Effect of *AGTPBP1* knockdown on drug resistance of A549 cells was visualized using Cell Counting Kit-8. The drugs used were doxorubicin and cisplatin. All values are expressed as mean ± standard deviation (SD) of at least three independent experiments, and statistical significance was analyzed using the two-tailed *t*-test. (*: *p* < 0.05; **: *p* < 0.01; ***: *p* < 0.001).

A distribution pattern of *AGTPBP1* expression in diverse normal tissues obtained from HPA [36] revealed that the mRNA expression level of *AGTPBP1* was higher in lung tissue and in bone marrow, cerebral cortex, granulocytes, and testis, as compared to that in other tissues (Supplementary Figure S1). To assess the role of *AGTPBP1* in lung cancer cell, we interfered *AGTPBP1* expression in A549 cells using *AGTPBP1*-targeted shRNA. RT-PCR analysis confirmed that sh*AGTPBP1* treatment decreased *AGTPBP1* expression by approximately 80% (Figure 1a). We compared cancer cell growth between control and *AGTPBP1-*silenced A549 cells for five days. The number of surviving cells was significantly increased by *AGTPBP1* knockdown at days 2, 3, 4, and 5 (Figure 1b). Cell migration assay revealed that the wound closure rate was significantly higher in *AGTPBP1-*silenced A549 cells at 24, 48, and 72 h (Figure 1c). In addition, the spheres formed by *AGTPBP1*-silenced cells were larger than those formed by control cells (Figure 1d). As sphere formation reflects the self-renewal capacity of tumor cells, we analyzed stemness marker genes, *SOX2*, *OCT4*, *NANOG*, and *c-MYC*. Among these, *SOX2* and *NANOG* were significantly upregulated in *AGTPBP1*-silenced A549 cells, as compared to control, whereas no differential expression of *OCT4* and *c-MYC* was observed between *AGTPBP1*-silenced cells and control cells (Figure 1e). Moreover, drug resistance of *AGTPBP1*-silenced A549 cells was intensified by treatment with the two commonly used cancer chemotherapeutic drugs, doxorubicin and cisplatin (Figure 1f). Overall, knockdown of *AGTPBP1* enhanced the oncogenic characteristics, including proliferation, migration, self-renewal, and drug-resistance, of A549 lung cancer cells, suggesting that *AGTPBP1* had a tumor-suppressing ability and could modulate the progression of LUAD.

### 3.2. Analysis of AGTPBP1 mRNA Expression Pattern in Lung Cancer

**Figure 2:**
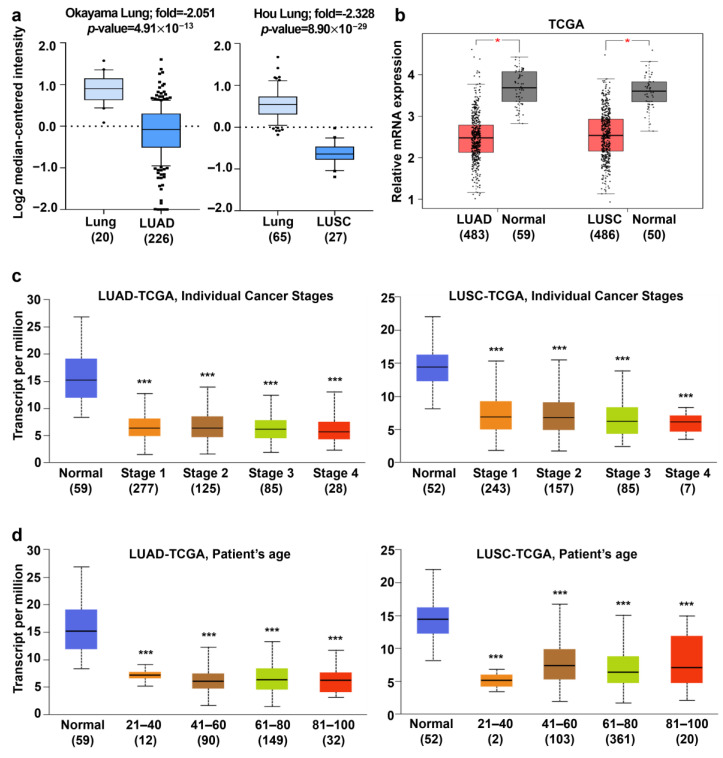
mRNA expression level of ATP/GTP binding protein 1 (*AGTPBP1*) in human lung cancer tissues and normal tissues. (**a**) mRNA level of *AGTPBP1* was downregulated in LUAD and LUSC, as determined from the Oncomine database. (**b**) The expression levels of *AGTPBP1* in LUAD or LUSC and normal tissue counterparts from The Cancer Genome Atlas (TCGA) database were compared using Gene Expression Profiling Interactive Analysis (GEPIA). (**c**,**d**) mRNA expression level of *AGTPBP1* according to the stage of cancer and patient’s age in LUAD and LUSC as compared to adjacent normal tissues. Statistical significance was determined using fold-change threshold >2 in the Oncomine database and *p-*value threshold < 0.05 in all databases. LUAD, lung adenocarcinoma; LUSC, lung squamous cell carcinoma (*: *p* < 0.05; ***: *p* < 0.001).

The tumor-suppressive effects of *AGTPBP1* suggest that the expression of *AGTPBP1* is reduced during lung oncogenesis. To examine the expression of *AGTPBP1* in lung cancer tissues and its adjoining normal tissues, datasets from Oncomine and TCGA databases were utilized. These included the Okayama dataset [37] with 20 normal lung tissues and 226 LUAD, and the Hou dataset [38] with 65 normal lung tissues and 27 LUSC. In both datasets, mRNA expression of *AGTPBP1* was significantly downregulated in lung carcinomas (Figure 2a). In the TCGA data from GEPIA web tool, *AGTPBP1* expression was downregulated in two types of NSCLC, as compared to its normal tissue counterparts (Figure 2b). To investigate the correlation between the mRNA level of *AGTPBP1* and the clinicopathological characteristics of lung cancers, we analyzed TCGA datasets using the UALCAN tool. Interestingly, *AGTPBP1* expression was upregulated independently of the stage of cancer (1–4) and patient age (21–40, 41–60, 61–80, and 81–100). *AGTPBP1* expression was significantly downregulated regardless of the stage of cancer in both LUAD and LUSC (Figure 2c). Moreover, *AGTPBP1* expression was significantly downregulated in the lungs of patients with LUAD and LUSC of all age groups as compared to those with normal lung tissues (Figure 2d). Analysis of other clinicopathological characteristics, including the race of the patient (Caucasian, African-American, and Asian), gender (male and female), nodal metastasis status (0–3), smoking habit, TP53 mutation status, and histological subtypes, showed that the mRNA level of *AGTPBP1* was significantly lower in LUAD and LUSC patients regardless of clinicopathological characteristics (Supplementary Figures S2 and S3). 

### 3.3. Analysis of AGTPBP1 Protein Level in Lung Cancer

**Figure 3:**
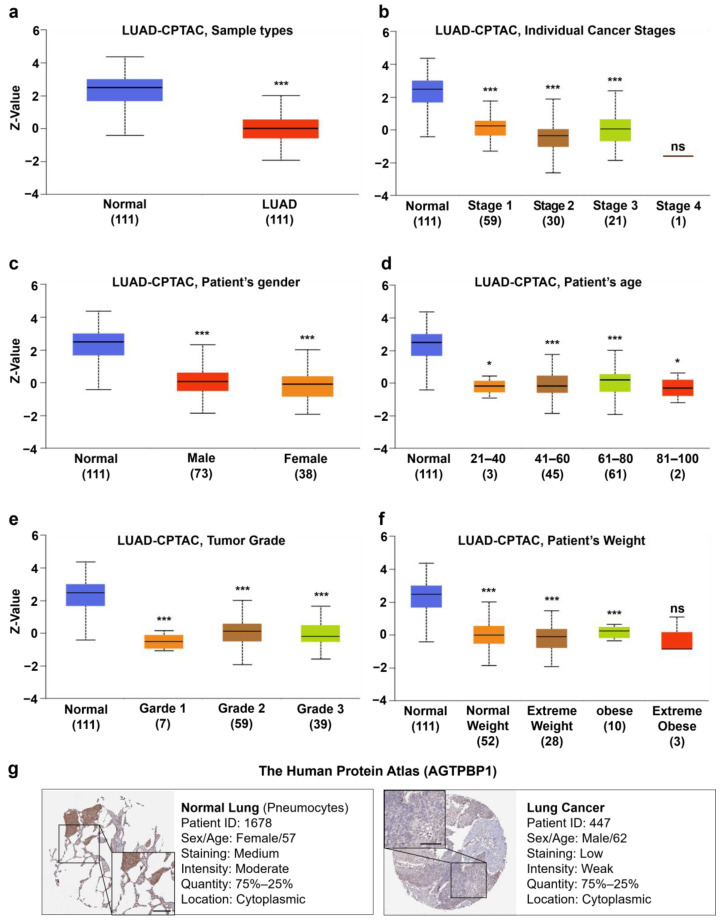
ATP/GTP binding protein 1 (*AGTPBP1*) protein expression profile in lung cancer tissues and adjacent normal tissues. (**a**–**f**) Box plot of protein expression level of *AGTPBP1* in LUAD (plotted in different colors) and normal lung tissues (plotted in blue) based on characteristics of patients with LUAD was plotted using the UALCAN web tool. The Clinical Proteomic Tumor Analysis Consortium (CPTAC) dataset was categorized as normal versus (**a**) LUAD, (**b**) cancer stage, (**c**) patient’s gender, (**d**) patient’s age, (**e**) tumor grade, and (**f**) patient’s weight. (**g**) Immunohistochemistry data of *AGTPBP1* with monoclonal antibody HPA057208 from the Human Protein Atlas (HPA) database. Pneumocytes from normal lung tissue of patient ID 1678 were moderately stained (left panel), whereas lung cancer tissue from patient ID 447 was stained low for *AGTPBP1* (right panel). (ns: non-significant; *: *p* < 0.05; ***: *p* < 0.001). LUAD, lung adenocarcinoma

To evaluate the protein expression level of *AGTPBP1*, we accessed the CPTAC dataset using UALCAN. In the CPTAC dataset, *AGTPBP1* protein expression was downregulated in primary LUAD regardless of the clinicopathological characteristics, including cancer stage, the patient’s race, gender, age, weight, and tumor grade (Figure 3a–f). Moreover, we observed that 3 out of 12 lung cancer tissues, including that of patient ID 447, showed a low level of staining for *AGTPBP1*, whereas immunohistochemistry data from HPA showed moderate cytosolic staining for *AGTPBP1* in normal lung tissues (Figure 3g). Altogether, these results indicated that the mRNA and protein expression levels of *AGTPBP1* were lower in lung cancer tissues, as compared to its neighboring normal tissues.

### 3.4. Analysis of Mutation and Copy Number Alterations of AGTPBP1 in Lung Cancer 

**Figure 4:**
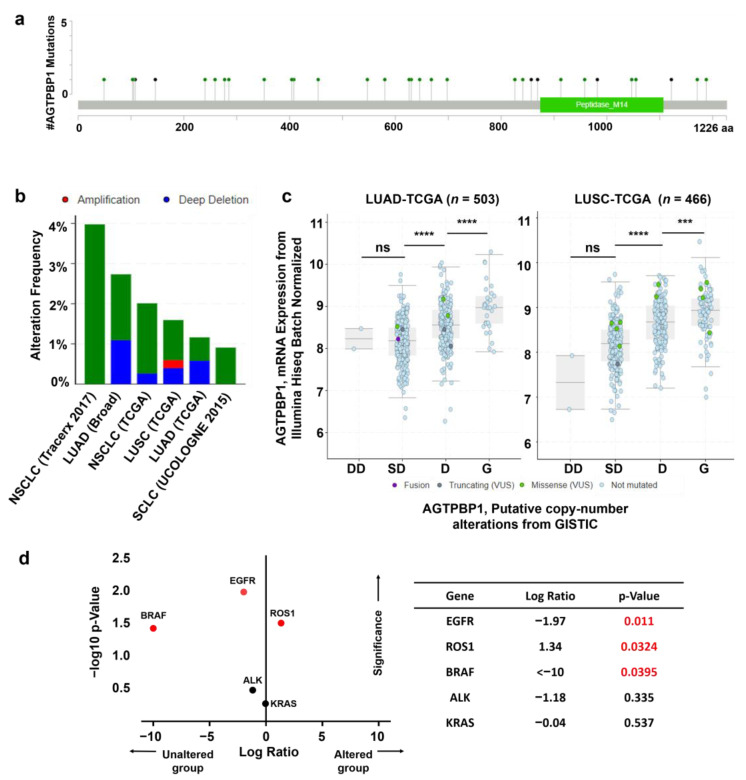
Mutation and copy number alterations (CNAs) of ATP/GTP binding protein 1 (*AGTPBP1*) in lung cancer, as determined using the cBioPortal. (**a**) Mutation diagram of protein domains between amino acids 0 and 1266 of *AGTPBP1*. (**b**) Alteration frequency of *AGTPBP1* obtained from 17 lung cancer studies with a total of 4624 samples. The sample threshold was set >100 and the alteration frequency was > 0.5%. (**c**) Correlation between *AGTPBP1* expression and CNAs in LUAD (*n* = 503) and LUSC (*n* = 466) in TCGA PanCancer dataset. LUAD, lung adenocarcinoma; LUSC, lung squamous cell carcinoma; DD, deep deletion; SD, shallow deletion; D, diploid; G, gain. (ns: non-significant; ***: *p* < 0.001; ****: *p* < 0.0001). (**d**) Co-occurrence pattern of mutation between *AGTPBP1* and some relevant biomarkers, *EGFR*, *ROS1*, *BRAF*, *ALK*, and *KRAS*. Log2-based ratio of mutated gene in *AGTPBP1* altered group to unaltered group was expressed along the *x*-axis and log10 *p*-values was expressed along the *y*-axis. Red—colored dots indicate genes with significant co-occurrence with *p*-value less than 0.05. NSCLC: non-small cell lung carcinoma, SCLC: small cell lung carcinoma, TCGA: The Cancer Genome Atlas.

To identify the alteration frequency of *AGTPBP1* gene in LUAD, we analyzed mutations and CNAs of *AGTPBP1* in a cohort of 4268 patients with lung cancer using the cBioPortal web. A total of 38 mutations in the *AGTPBP1* gene were identified among lung cancer samples; the mutations were evenly distributed in all regions, including zinc carboxypeptidase, which belongs to the M14 peptidase family with 874–1107 amino acids (Figure 4a). Moreover, the alteration frequency of *AGTPBP1* was 4% in the TRAcking Cancer Evolution through therapy (TRACERx) dataset and 3% in the Broad Institute dataset. Deep deletion was predominantly appeared in approximately 0.2–1% of the patients in the Broad Institute and TCGA datasets (Figure 4b). CNAs in LUAD and LUSC were significantly correlated with *AGTPBP1* expression. Moreover, a significant proportion of patients exhibited shallow deletion of *AGTPBP1,* and significantly lower *AGTPBP1* expression as compared to those with diploid CNA status (Figure 4c). These data suggested that shallow deletion of *AGTPBP1* could be partially responsible for the reduced *AGTPBP1* expression in lung cancer. Co-occurrence of mutation in *AGTPBP1* and other altered biomarkers *EGFR, KRAS, BRAF, ALK* and *ROS1* in lung cancer was analyzed (Figure 4d). *ROS1* alteration patient samples were significantly counted in the *AGTPBP1* altered group, whereas *EGFR* and *BRAF* mutated patients was predominant in *AGTPBP1* unaltered group. Overall, these results suggested that alteration of *AGTPBP1* could be associated with lung cancer.

### 3.5. Analysis of Correlation between AGTPBP1 Expression and Patient Survival 

**Figure 5:**
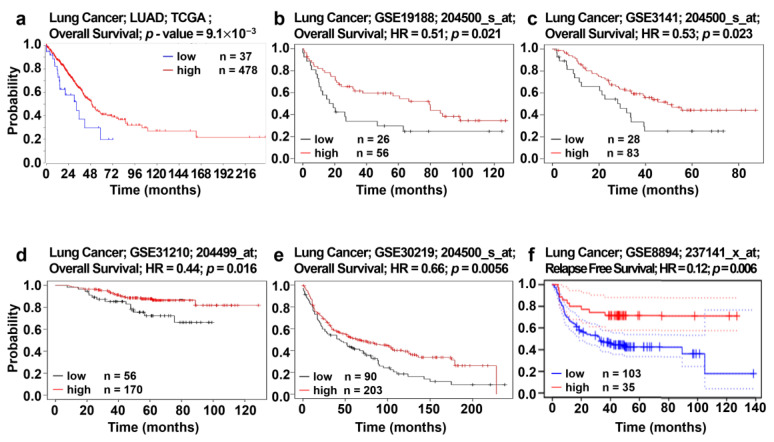
Kaplan-Meier survival curves for lung cancer patients according to ATP/GTP binding protein 1 (*AGTPBP1*) expression in non-small cell lung carcinoma (NSCLC). (**a**) Survival curve comparing high and low *AGTPBP1* expression groups of in LUAD datasets of TCGA (LUAD-TCGA) from R2 database. (**b**–**e**) Overall survival curves of NSCLC in four different lung cancer datasets, including GSE19188 (*n* = 82), GSE3141 (*n* = 111), GSE31210 (*n* = 226), and GSE30219 (*n* = 293) in Kaplan-Meier plotter. (**f**) Relapse free survival pattern of the NSCLC cohort GSE8894 (*n* = 138) in PrognoScan. Statistical significance was determined using *p-*value threshold < 0.05 in all databases. HR: hazard ratio, TCGA: The Cancer Genome Atlas, LUAD: lung adenocarcinoma.

The effects of *AGTPBP1* knockdown suggest the suppressive role of *AGTPBP1* in tumor progression. Therefore, we analyzed the relationship between *AGTPBP1* expression and prognosis in lung cancer using gene expression datasets. The association between *AGTPBP1* expression and the survival of patients with lung cancer was examined using R2: Genomics Analysis and Visualization Platform, KM-plotter, and PrognoScan database. The LUAD dataset in TCGA database was analyzed using R2: Genomics Analysis and Visualization Platform. In the LUAD-TCGA dataset, *AGTPBP1* expression positively correlated with overall survival (Figure 5a). To examine the relationship between patient survival and *AGTPBP1* expression in other datasets, we utilized the KM-plotter and analyzed results using univariate analysis with a *p*-value threshold < 0.05. Four different datasets, including GSE19188 [38], GSE3141 [39], GSE31210 [37], and GSE30219 [40], also displayed positive correlation between overall survival and *AGTPBP1* expression (Figure 5b–e). Additionally, in the GSE8894 [41] dataset of PrognoScan web tool, *AGTPBP1* expression level positively correlated with relapse-free survival (Figure 5f). Therefore, *AGTPBP1* expression showed a significant positive correlation with patient survival in multiple datasets of patients with NSCLC, suggesting a tumor-suppressive role of *AGTPBP1*.

### 3.6. Analysis of Genes Co-Expressed with AGTPBP1 in Lung Cancer 

**Figure 6:**
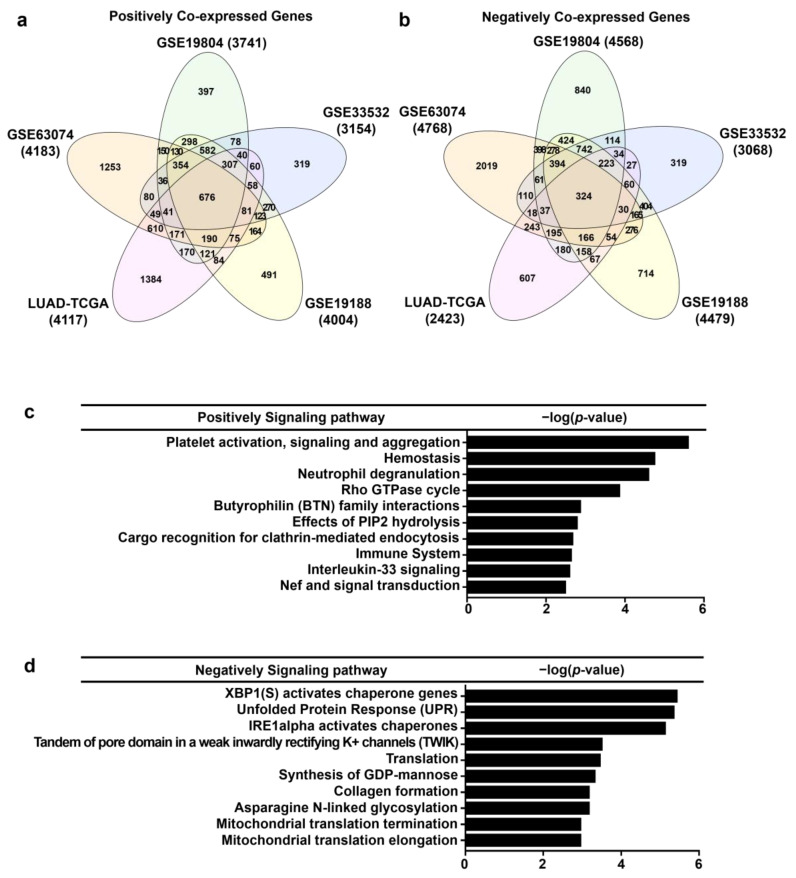
Positively and negatively correlated genes and signaling pathways in five public datasets. (**a**,**b**) Venn diagram of genes positively and negatively correlated with ATP/GTP binding protein 1 (*AGTPBP1*) was acquired using InteractiVenn. (**c**,**d**) List of top 10 signaling pathways from positively and negatively co-expressed genes obtained from the Reactome database. Statistical significance was determined using a *p-*value threshold < 0.01.

To elucidate the potential signaling mechanism related to *AGTPBP1* expression in lung cancer, we acquired the correlation gene sets from the following five different transcriptome datasets of lung cancer using the R2 data tool: GSE63074 [42], GSE19804 [43], GSE33532 [44], GSE19188 [38], and LUAD-TCGA. A total of 676 co-expressed genes were positively co-altered (Figure 6a) and 324 co-expressed genes were negatively co-altered (Figure 6b) with *AGTPBP1* in the five selected datasets. Reactome pathway analysis indicated that the positively co-altered genes with *AGTPBP1* were mainly involved in signaling pathways associated with the immune microenvironment, including coagulation, the innate system, butyrophilin (BTN) family interaction, interleukin (IL)-33, and Nef-related signaling. Some positively correlated genes were categorized to the process of endocytosis, including cargo recognition in clathrin-mediated endocytosis and phosphatidylinositol phosphate 2 hydrolysis (Figure 6c). Besides, the negatively correlated genes were mainly involved in protein folding during endoplasmic reticulum (ER) stress, including activation of X-box binding protein 1 (XBP1), inositol-requiring enzyme 1 (IRE1), activated chaperones, and a tandem of pore domains in a weak inwardly rectifying K+ channels (TWIK). A few of them were involved in glycolytic functions and mitochondrial functions, including elongation and termination of mitochondrial translation (Figure 6d). These results suggested that *AGTPBP1* could be linked to certain key pathways related to immune microenvironment regulation and protein processing in oncogenic pathways.

### 3.7. Correlation Anlaysis between Immune Infiltration and AGTPBP1 Expression in LUAD

**Figure 7:**
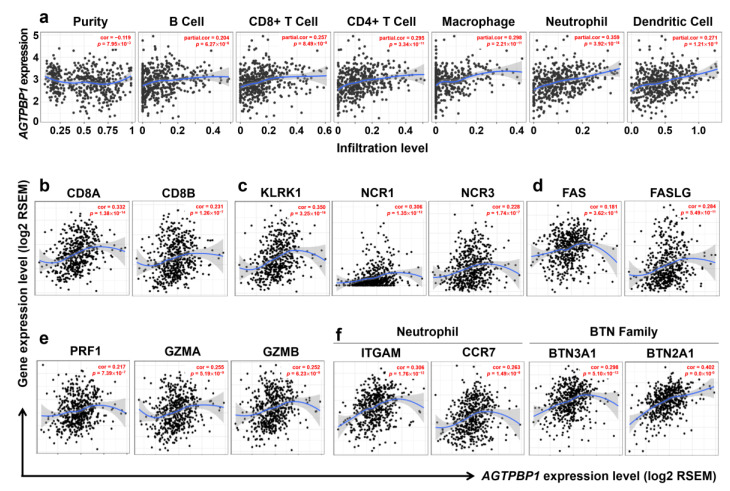
Correlation of ATP/GTP binding protein 1 (*AGTPBP1*) expression with diverse immune cells and related marker genes in lung adenocarcinoma (LUAD), analyzed using the Tumor Immune Estimation Resource (TIMER) web tool (*n* = 515). (**a**) Correlation between tumor purity and levels of diverse tumor-infiltrating immune cells related to *AGTPBP1* expression. Correlation of *AGTPBP1* expression with cytotoxicity marker genes induced by effector cells: (**b**) CD8^+^ T cell markers, including *CD8A* and *CD8B*; (**c**) gene markers of natural killer (NK) cell activation receptors, including killer cell lectin like receptor K1 (*KLRK1*), natural cytotoxicity triggering receptor 1 (*NCR1*), and natural cytotoxicity triggering receptor 3 (*NCR3*); (**d**) death signal marker genes, including Fas cell surface death receptor (*FAS*) and Fas ligand (*FASLG*); and (**e**) gene markers of cytolytic molecules, including perforin (*PRF1*), granzyme A (*GZMA*), and granzyme B (*GZMB*). (**f**) Positive correlation between *AGTPBP1* expression and neutrophil and butyrophilin (BTN) family gene markers. Correlation constants and *p-*values are listed in Table 1, Table 2 and Table 3. ITGAM: integrin alpha M, CCR7: C-C chemokine receptor type 7.

Ontology analysis of genes co-altered with *AGTPBP1* suggested that *AGTPBP1* expression could be involved in the regulation of the tumor immune microenvironment; therefore, we investigated the relationship between immune cell infiltration and *AGTPBP1* expression using DICE database and TCGA data from TIMER web tool. DICE database analysis revealed that the transcriptional level of *AGTPBP1* was significantly upregulated in natural killer (NK) cells and classical monocytes (Supplementary Figure S4). In the TCGA dataset, *AGTPBP1* expression negatively correlated with tumor purity in LUAD (cor. = −0.119, *p* = 7.95 × 10^−3^), indicating that *AGTPBP1* was probably expressed by the tumor-infiltrating immune cells. Higher *AGTPBP1* expression in LUAD tissues notably increased the infiltrated level of certain types of immune cells, including B cells, CD8^+^ T cells, CD4^+^ T cells, macrophages, neutrophils, and dendritic cells (DCs), whereas AGTPBP1 expression in LUSC was not significantly correlated with tumor purity and the infiltration of macrophages and neutrophils (Figure 7a and Table 1). To further investigate the correlation between *AGTPBP1* mRNA expression and diverse subsets of tumor-infiltrating immune cells in LUAD, we carried out correlation analysis using markers of T cells, B cells, monocytes, M1 and M2 macrophages, neutrophils, NK cells, and DCs (Table 2). In the TIMER database, *AGTPBP1* expression significantly correlated with several immune cell markers in LUAD with or without purity adjustment. Moreover, the correlation between *AGTPBP1* expression and diverse immune cell markers in LUSC was significant, excluding regulatory T cell (Treg) and exhausted T cell signatures. In this context, *AGTPBP1* expression exhibited relatively high correlation with specific gene marker of CD8^+^ T cells, monocytes, tumor-associated macrophages, M1 and M2 macrophages, Tregs, and exhausted T cells in LUAD (Table 2). 

The infiltration of anti-tumor effector cells i.e., CD8^+^ T cells was analyzed using CD8A and CD8B as gene markers (Figure 5b). NK cells effectively inhibit the function of cancer cells through activation of killer cell lectin like receptor K1 (*KLRK1*) and natural cytotoxicity triggering receptors 1 and 2 (*NCR1* and *NCR2*) [45]. In addition, NK cells induce the expression of FasL/Fas (*FAS*/*FASLG*), leading to apoptosis of tumor cells [46], and also affect tumor cells via multiple approaches, including direct lysis by perforin 1 (*PRF1*) and granzyme A and B (*GZMA* and *GZMB*) [47]. Importantly, all these markers exhibited significant positive correlation with *AGTPBP1* expression (Figure 7c–e, and Table 2). Moreover, we also analyzed the marker genes of neutrophils and butyrophilin (BTN) family using the Reactome webtool (Figure 6c). The neutrophil markers integrin alpha M (*ITGAM*; *CD11b*) and *CCR7* positively correlated with *AGTPBP1* expression (Table 2); the BTN family markers *BTN3A1* (cor. = 0.298, *p* = 5.10 × 10^−12^) and *BTN2A1* (cor. = 0.402, *p* = 0), which are essential for human γδ T cell recognition, significantly correlated with *AGTPBP1* expression (Figure 7e) [48]. Altogether, these findings indicated that *AGTPBP1* expression correlated with immune cell infiltration signatures in LUAD, suggesting a prognostic value of *AGTPBP1* owing to its association with the immune microenvironment in LUAD.

## 4. Discussion

*AGTPBP1* encodes a zinc carboxypeptidase that mediates the deglutamylation of target proteins, including tubulins and myosin light chain kinase [1,6]. Mutations in human and mouse *AGTPBP1* genes are closely related to childhood-onset neurodegeneration [6]. However, the expression and function of *AGTPBP1* in malignancy has not been comprehensively investigated yet. Human lung tissues express relatively high levels of *AGTPBP1* mRNA as compared to other tissue types, strongly suggesting the role of AGTPBP1 in lung function and related diseases. In this study, we provided evidence for the potential function of AGTPBP1 and its clinical association with lung cancer. 

We found that reduced *AGTPBP1* expression by knockdown in lung cancer cells increased the oncogenic characteristics, including proliferation, migration, sphere formation, and drug resistance, of cells in vitro. Moreover, *AGTPBP1* expression was downregulated in lung cancer tissues, as compared to their adjacent normal lung tissues in NSCLC. CNA analysis showed that a significant proportion of the lung cancer tissues displayed shallow deletion, and *AGTPBP1* expression was significantly reduced in cells with shallow deletion as compared to diploid cells. We also analyzed the relationship between *AGTPBP1* expression and patient survival rate in various lung cancer datasets using web-based analysis tools, including KM-plotter and PrognoScan. Association of lower *AGTPBP1* expression with poor prognosis suggested a diagnostic value of *AGTPBP1* in lung cancer. These results suggested that lower expressions of *AGTPBP1*, which may be partially caused by CNA in lung cancer, could accelerate the oncogenic properties of cancer cells, resulting in poor prognosis. 

Furthermore, we investigated the possible pathways associated with *AGTPBP1* in lung cancer by analyzing the genes co-expressed with *AGTPBP1* using five different datasets. Ontology analysis explained that the positively correlated genes were associated with the regulation of the immune microenvironment. As the tumor infiltrating of immune cells influences the outcome of cancer by altering the balance of suppressive versus promotive tumor microenvironment [49], this result suggests the important role of *AGTPBP1* in immune microenvironment regulation. The positively correlated ontology term “neutrophil degranulation” indicated an association between *AGTPBP1* and immune microenvironment regulation in lung cancer as T cell proliferation mediated by tumor-associated neutrophils is augmented in a positive-feedback loop in the earliest stages of lung cancer n [50]. Furthermore, IL-33 has been reported to significantly modulate the tumor microenvironment by recruiting immune cells in lung carcinogenesis, both in vitro and in vivo [51]. Our study showed that genes co-expressed with *AGTPBP1* were associated with Nef genes of human immunodeficiency virus (HIV) and signal transduction, which antagonize the chemokine receptor CXCR4 and have an apoptotic effect on human colorectal cancer [52]. Additionally, *AGTPBP1* is known to play an essential role in T lymphocyte development in zebrafish [53], suggesting the important role of *AGTPBP1* in the control of the tumor immune microenvironment in lung cancer. 

We further evaluated the association of *AGTPBP1* with immune cell infiltration and cytotoxicity markers in lung cancer using the TIMER webtool. *AGTPBP1* expression positively correlated with signature genes of various subsets of immune cells in LUAD. Macrophage and neutrophil infiltration exhibited a relatively high correlation with *AGTPBP1* expression in the immune microenvironment in LUAD. Furthermore, we demonstrated that *AGTPBP1* could improve patient outcomes owing to infiltration and cytotoxicity activity of CD8^+^ T cells and NK cells. Previous studies have reported that infiltrating CD8^+^ T cells and NK cells in NSCLC suppresses cancer progression and could be indicators of favorable prognosis [54]. NK cells mediate several effector functions and include the following: (1) direct cytotoxicity through exocytosis of cytotoxic granules containing perforins and granzyme B; (2) apoptosis of target cells via death receptor; and (3) production of immune-active cytokines, including IFN-γ, TNF-α, and GM-CSF [54]. The cytotoxicity and cytolytic markers of CD8^+^ T cells and NK cells positively correlated with *AGTPBP1* expression in LUAD. We also demonstrated that BTN family members play cytotoxic roles in tumor cells via interaction with γδ T cells [48]. γδ T cells are known as attractive effector cells for cancer immunotherapy as they secrete cytokines and exhibit cytotoxicity against a wide range of cancer cells [55]. Our results suggested that lower expressions of *AGTPBP1* were associated with low cytotoxicity in LUAD, and *AGTPBP1* might be a prognostic factor for lung cancer. 

However, co-expression analysis demonstrates that *AGTPBP1* expression is negatively correlated with the expression of genes associated with ER stress-related pathways. It has been reported that ER stress is involved in the degeneration of Purkinje cells [56], which is associated with *AGTPBP1* expression. There are three main ER stress-signaling branches involved in tumorigenesis, which include IRE1, activating transcription factor 6 (ATF6), and pancreatic ER kinase-like ER kinase (PERK) [57]. Among these, IRE1–XBP1 signaling is increased in many human cancers, including breast cancer, hepatocellular carcinoma, and pancreatic adenocarcinoma [58]. Higher level of XBP1 correlates with lower survival rate and poor prognosis of patients with glioblastoma; conversely, ovarian cancer mouse treated with XBP1-silencing nanoparticles exhibited better prognosis, as compared to control [59,60]. Altogether, our findings indicated that the low expression of *AGTPBP1* was associated with high ER stress in tumors, which might be related to poor outcomes in patients with lung cancer. 

Therefore, these results suggest that *AGTPBP1* expression impacts on lung cancer suppression by controlling tumor cell properties and the immune microenvironment. CCP1 is the first member of subfamily cytosolic carboxypeptidase (CCP), which processes tubulin with polyglutamylation and affects its stability [61]. Some types of microtubule-targeted drugs, such as paclitaxel and the *Vinca* alkaloids is used as anti-cancer therapeutics, since tubulin stability is significantly important in the process of mitosis, which is main objectives in clinical cancer investigation [62]. Furthermore, Das, Viswanath et al. alluded to a crucial role of polyglutamylation in tumorigenesis and cancer cell resistance [14]. Polyglutamylation contributes negative charge to the C-terminal that is required on neuronal differentiation, but increased abundance leads to both carcinogenesis and chemo-resistance [63]. Therefore, modulation of *AGTPBP1* expression could be a potential therapeutic approach for lung cancer. 

Doxorubicin and cisplatin were widely used anti-cancer drugs. In lung cancers, cisplatin is frequently used with the combination of other drugs in platinum doublet or triplet regimens [64]. Doxorubicin combinations or encapsulation with nanoparticles were also sought to enhance the anti-tumor activity and reduced toxicity in lung cancers [65,66,67,68]. Enhanced survival of *AGTPBP1* knockdown cell in the single use of doxorubicin and cisplatin treatment in Figure 1f meant that *AGTPBP1* function could be involved in death-inducing mechanism such as oxidative stress, which is induced by both doxorubicin and cisplatin in A549 cells [69,70]. However, drug sensitivity tests with more frequently used regimes such as platinum-pemetrexed, platinum-taxol or platinum-gemcitabine combination [64] could provide the stronger clinical meaning accessing patient survival. 

Advances in DNA sequencing technology revealed the driver mutations in lung cancers at the genes including *EGFR*, *ROS1*, *BRAF*, *ALK*, and *KRAS* [71,72,73]. Detecting the driver mutation is essential to determine the appropriate targeted therapy. A significant association of mutations in some relevant genes, including *BRAF*, *EGFR*, and *ROS1* with *AGTPBP1* (Figure 4d), suggested the possible combinatorial effect of mutations in *AGTPBP1* and other driver genes, which should be perused in further study. 

We only used one lung cancer cell line, A549, with *KRAS* mutation [74]. Activating KRAS mutation rate is around 10–30% in human lung adenocarcinoma [75,76,77]. However, suppressive effect of *AGTPBP1* expression on lung adenocarcinoma was suggested by the analysis of LUAD-TCGA datasets without considering *KRAS* mutation. Moreover, *KRAS* mutation rate was not significantly associated with *AGTPBP1* (Figure 4d). A differential effect of AGTPBP1 expression on the genetical subtypes of lung cancer could be investigated using additional lung cancer cell lines without KRAS mutation such as HCC78 [78] and EBC-1 [79] in future study. In addition, in vivo studies must be carried out in the future to clearly understand the mechanisms underlying the role of *AGTPBP1* in lung cancer.

## 5. Conclusions

In this multidimensional analysis of *AGTPBP1* expression in lung cancer database and in vitro with a cancer cell line, we suggested the first evidence of the correlation between the *AGTPBP1* expression and clinical outcomes in lung cancer. Our systematic analysis reveals the prognostic value of *AGTPBP1* expression and suggests potential *AGTPBP1*-related mechanisms in lung cancer progression, which include the effects on oncogenic properties in tumor cells and tumor immune microenvironments. Thus, our study contributes an overall understanding of the therapeutic role of *AGTPBP1* in lung cancer and the possible therapeutic use for the cure of lung cancer patients.

## Figures and Tables

**Table 1:** Analysis of correlation between ATP/GTP binding protein 1 (*AGTPBP1)* expression and immune infiltration in non-small cell lung carcinoma (NSCLC).

Description	LUAD		LUSC	
	Cor	*p*	Cor	*p*
Purity	−0.119	*	0.069	0.133
B Cell	0.204	***	0.135	*
CD8^+^ T Cell	0.257	***	0.144	*
CD4^+^ T Cell	0.295	***	0.132	*
Macrophage	0.298	***	0.063	0.169
Neutrophil	0.359	***	0.072	0.114
Dendritic Cell	0.271	***	0.141	*

LUAD, lung adenocarcinoma; LUSC, lung squamous cell carcinoma; Cor, Spearman’s rho value. *: *p* < 0.01; ***: *p* < 0.0001.

**Table 2:** Analysis of correlation between ATP/GTP binding protein 1 (*AGTPBP1*) and immune cell gene markers using tumor immune estimation resource (TIMER) database.

Description	Gene Marker	LUAD				LUSC			
		None		Purity		None		Purity	
		Cor	*p*	Cor	*p*	Cor	*p*	Cor	*p*
CD8^+^ T cell	*CD8A*	0.332	***	0.339	***	0.161	**	0.158	**
	*CD8B*	0.231	***	0.233	***	0.126	*	0.113	0.014
T cell (general)	*CD3D*	0.180	***	0.185	***	0.092	0.040	0.090	0.050
	*CD3E*	0.293	***	0.305	***	0.127	*	0.127	*
	*CD2*	0.260	***	0.269	***	0.128	*	0.121	*
B cell	*CD19*	0.184	***	0.199	***	0.106	0.018	0.110	0.017
	*CD79A*	0.131	*	0.147	*	0.089	0.046	0.094	0.040
Monocyte	*CD86*	0.331	***	0.331	***	0.154	**	0.143	*
	*CD115 (CSF1R)*	0.303	***	0.303	***	0.150	**	0.139	*
TAM	*CCL2*	0.179	***	0.186	***	0.066	0.141	0.063	0.168
	*CD68*	0.252	***	0.261	***	0.088	0.048	0.087	0.058
	*IL10*	0.278	***	0.280	***	−0.002	0.965	0.003	0.947
M1 Macrophage	*NOS2*	0.262	***	0.262	***	0.108	0.016	0.119	*
	*IRF5*	0.267	***	0.277	***	0.070	0.117	0.060	0.188
	*COX2 (PTGS2)*	0.010	0.825	0.007	0.880	−0.075	0.095	−0.074	0.107
M2 Macrophage	*CD163*	0.408	***	0.408	***	0.142	*	0.134	*
	*VSIG4*	0.284	***	0.285	***	0.066	0.139	0.056	0.219
	*MS4A4A*	0.322	***	0.327	***	0.114	0.010	0.105	0.022
Neutrophil	*CEACAM8*	0.117	*	0.127	*	−0.021	0.642	−0.023	0.615
	*ITGAM*	0.306	***	0.316	***	0.190	***	0.179	***
	*CCR7*	0.263	***	0.275	***	0.169	**	0.171	**
NK cell	*KIR2DL1*	0.206	***	0.210	***	0.038	0.392	0.052	0.256
	*KIR2DL3*	0.235	***	0.240	***	0.054	0.231	0.035	0.445
	*KIR2DL4*	0.178	***	0.178	***	0.034	0.446	0.022	0.634
	*KIR3DL1*	0.208	***	0.196	***	0.089	0.046	0.077	0.093
	*KIR3DL2*	0.206	***	0.209	***	0.107	0.017	0.109	0.017
	*KIR3DL3*	0.159	**	0.156	**	0.064	0.152	0.075	0.103
	*KIR2DS4*	0.216	***	0.218	***	0.056	0.209	0.072	0.115
Dendritic cell	*HLA-DPB1*	0.155	**	0.161	**	0.122	*	0.112	0.014
	*HLA-DQB1*	0.078	0.078	0.083	0.066	0.062	0.164	0.055	0.229
	*HLA-DRA*	0.139	*	0.143	*	0.098	0.028	0.085	0.064
	*HLA-DPA1*	0.181	***	0.186	***	0.136	*	0.124	*
	*BDCA-1 (CD1C)*	0.066	0.133	0.070	0.121	0.046	0.305	0.041	0.367
	*BDCA-4 (NRP1)*	0.223	***	0.217	***	0.062	0.164	0.061	0.180
	*CD11c (ITGAX)*	0.377	***	0.383	***	0.176	***	0.175	**
Th1	*T-bet (TBX21)*	0.350	***	0.362	***	0.205	***	0.206	***
	*STAT4*	0.188	***	0.200	***	0.065	0.145	0.063	0.172
	*STAT1*	0.339	***	0.347	***	0.139	*	0.141	*
	*IFN-γ (IFNG)*	0.236	***	0.247	***	0.099	0.027	0.098	0.033
	*TNF-α (TNF)*	0.143	*	0.145	*	−0.044	0.322	−0.052	0.258
Th2	*GATA3*	0.261	***	0.273	***	−0.125	*	−0.136	*
	*STAT6*	0.151	**	0.167	**	0.074	0.099	0.074	0.106
	*STAT5A*	0.429	***	0.438	***	0.239	***	0.237	***
	*IL13*	0.088	0.046	0.104	0.021	0.202	***	0.202	***
Tfh	*BCL6*	0.204	***	0.208	***	0.223	***	0.233	***
	*IL21*	0.211	***	0.218	***	0.113	0.011	0.104	0.022
Th17	*STAT3*	0.251	***	0.249	***	0.208	***	0.215	***
	*IL17A*	0.101	0.022	0.108	0.017	0.035	0.440	0.031	0.493
Treg	*FOXP3*	0.222	***	0.232	***	0.154	**	0.146	*
	*CCR8*	0.315	***	0.326	***	0.200	***	0.193	***
	*STAT5B*	0.478	***	0.483	***	0.355	***	0.360	***
	*TGFβ (TGFB1)*	0.177	***	0.177	***	−0.161	**	−0.148	*
Exhausted T cell	*PD-1 (PDCD1)*	0.235	***	0.237	***	0.154	**	0.154	**
	*CTLA4*	0.290	***	0.301	***	0.168	**	0.164	**
	*LAG3*	0.241	***	0.248	***	0.130	*	0.122	*
	*TIM-3 (HAVCR2)*	0.311	***	0.313	***	0.141	*	0.128	*

LUAD, lung adenocarcinoma; LUSC, lung squamous cell carcinoma; TAM, tumor-associated macrophage; NK, natural killer; Th, T helper cell; Tfh, follicular helper T cell; Treg, regulatory T cell; Cor, Spearman’s rho value; “None” represents correlation without adjustment, and “Purity” represents correlation with purity adjustment. *: *p* < 0.01; **: *p* < 0.001; ***: *p* < 0.0001.

**Table 3:** Analysis of correlation between natural killer (NK) cell-mediated cytotoxicity marker genes and ATP/GTP binding protein 1 (*AGTPBP1*) expression using tumor immune estimation resource (TIMER) database.

Description	Gene Marker	LUAD				LUSC			
		None		Purity		None		Purity	
		Cor	*p*	Cor	*p*	Cor	*p*	Cor	*p*
Activation receptors	*KLRK1*	0.350	***	0.358	***	0.183	***	0.182	***
	*NCR1*	0.306	***	0.309	***	0.182	***	0.168	**
	*NCR2*	0.045	0.312	0.066	0.145	0.029	0.518	0.036	0.435
	*NCR3*	0.228	***	0.242	***	0.124	*	0.131	*
FAS/FASL	*FAS*	0.181	***	0.190	***	0.052	0.247	0.064	0.163
	*FASLG*	0.284	***	0.291	***	0.108	0.016	0.106	0.020
Cytolytic molecules	*GZMA*	0.255	***	0.257	***	0.053	0.238	0.050	0.271
	*GZMB*	0.252	***	0.256	***	0.105	0.019	0.106	0.020
	*PRF1*	0.217	***	0.221	***	0.126	*	0.127	*

LUAD, lung adenocarcinoma; LUSC, lung squamous cell carcinoma; Cor, Spearman’s rho value; “None” represents correlation without adjustment, and “Purity” represents correlation with purity adjustment. *: *p* < 0.01; **: *p* < 0.001; ***: *p* < 0.0001.

## Supplementary Materials

The following are available online at https://www.mdpi.com/2075-4426/10/4/263/s1, Supplementary Figure S1; Distribution of *AGTPBP1* expression in various normal tissue types through the HPA dataset, Supplementary Figure S2; The transcription level of *AGTPBP1* based on LUAD patient’s characteristics, Supplementary Figure S3; The transcription level of *AGTPBP1* based on LUSC patient’s characteristics, Supplementary Figure S4; *AGTPBP1* expression level in various immune cells using the DICE web tools, Supplementary Table S1; Primers in RT-PCR.
