# Supplementary material for: Expression of ATP/GTP Binding Protein 1 Has Prognostic Value for the Clinical Outcomes in Non-Small Cell Lung Carcinoma
Source: J Pers Med. 2020 Dec 2;10(4):263. doi: 10.3390/jpm10040263 (PMC7761608; doi:10.3390/jpm10040263)
Supplement: Supplementary file 1 [file jpm-10-00263-s001.tgz › Supplementary Figure_S4.docx]

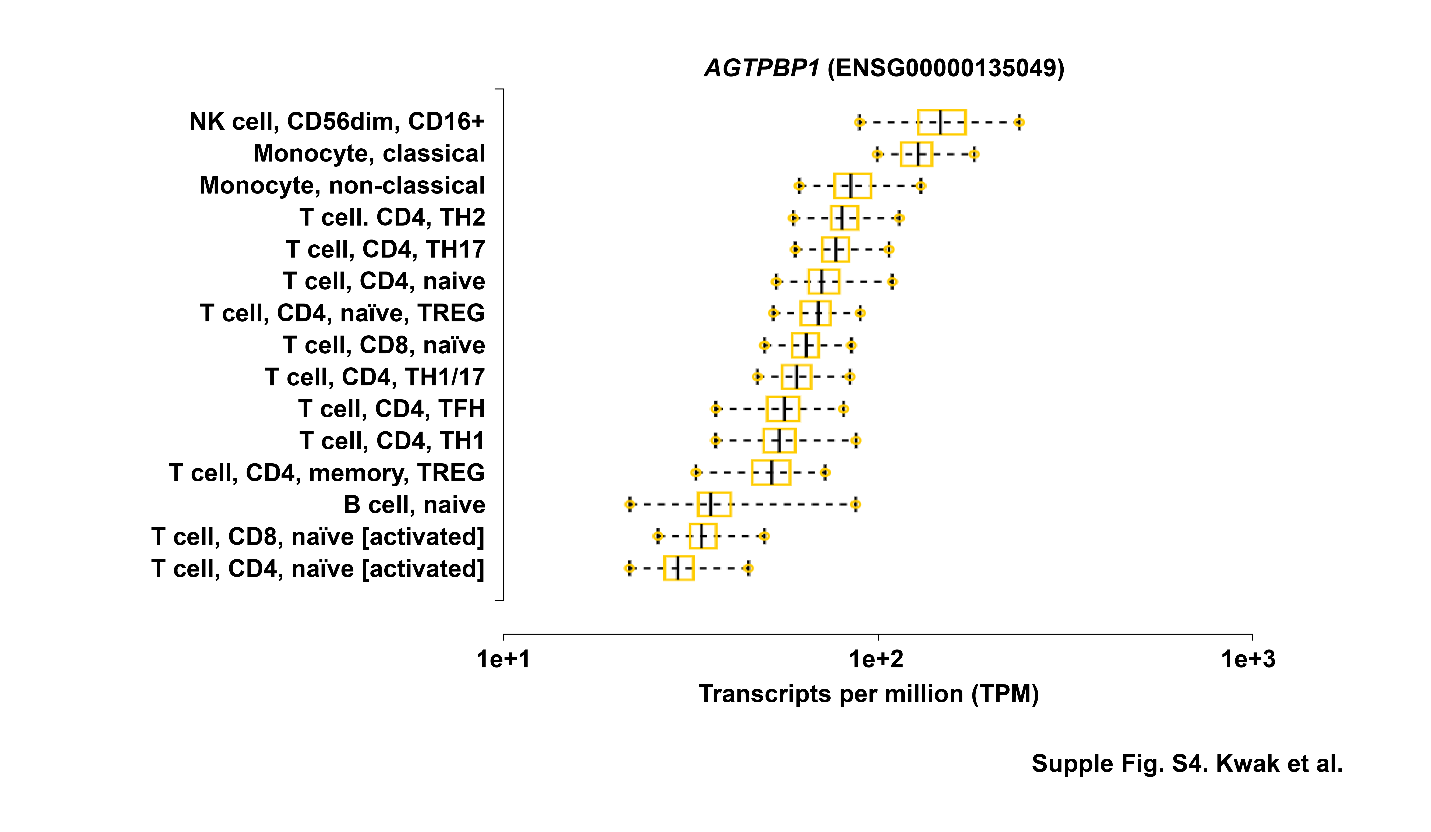


**Supplementary Figure S4**. *AGTPBP1* expression level in various immune cells using the Database of Immune Cell Expression, expression quantitative trait loci (eQTL), and epigenomics (DICE) web tools.
