# Supplementary material for: Expression of ATP/GTP Binding Protein 1 Has Prognostic Value for the Clinical Outcomes in Non-Small Cell Lung Carcinoma
Source: J Pers Med. 2020 Dec 2;10(4):263. doi: 10.3390/jpm10040263 (PMC7761608; doi:10.3390/jpm10040263)
Supplement: Supplementary file 1 [file jpm-10-00263-s001.tgz › Supplementary Table_S1.docx]

**Supplementary Table S1. Primers sequences for RT-PCR**

| **Primers** | **Forward (5'-3')** | **Reverse (5'-3')** |
| --- | --- | --- |
| *GAPDH* | AAT CCC ATC ACC ATC TTC CAG | CAC GAT ACC AAA GTT GTC ATG |
| *AGTPBP1* | GCA GTG AAG CGT TTA CCC T | GCT GGG GCG ATA TGG CTC |
| *SOX2* | ACA CCA ATC CCA TCC ACA CT | GCA AGA AGC CTC TCC TTG AA |
| *OCT4* | GTC CCA GGA CAT CAA AGC TC | CTC CAG GTT GCC TCT CAC TC |
| *NANOG* | GTC TTC TGC TGA GAT GCC TCA CA | CTT CTG CGT CAC ACC ATT GCT AT |
| *c-MYC* | TCC ACG AAA CTT TGC CCA TAG | AGT AGA AAT ACG GCT GCA CC |
