# Supplementary material for: Expression of ATP/GTP Binding Protein 1 Has Prognostic Value for the Clinical Outcomes in Non-Small Cell Lung Carcinoma
Source: J Pers Med. 2020 Dec 2;10(4):263. doi: 10.3390/jpm10040263 (PMC7761608; doi:10.3390/jpm10040263)
Supplement: Supplementary file 1 [file jpm-10-00263-s001.tgz › Supplementary Figure_S1.docx]

**
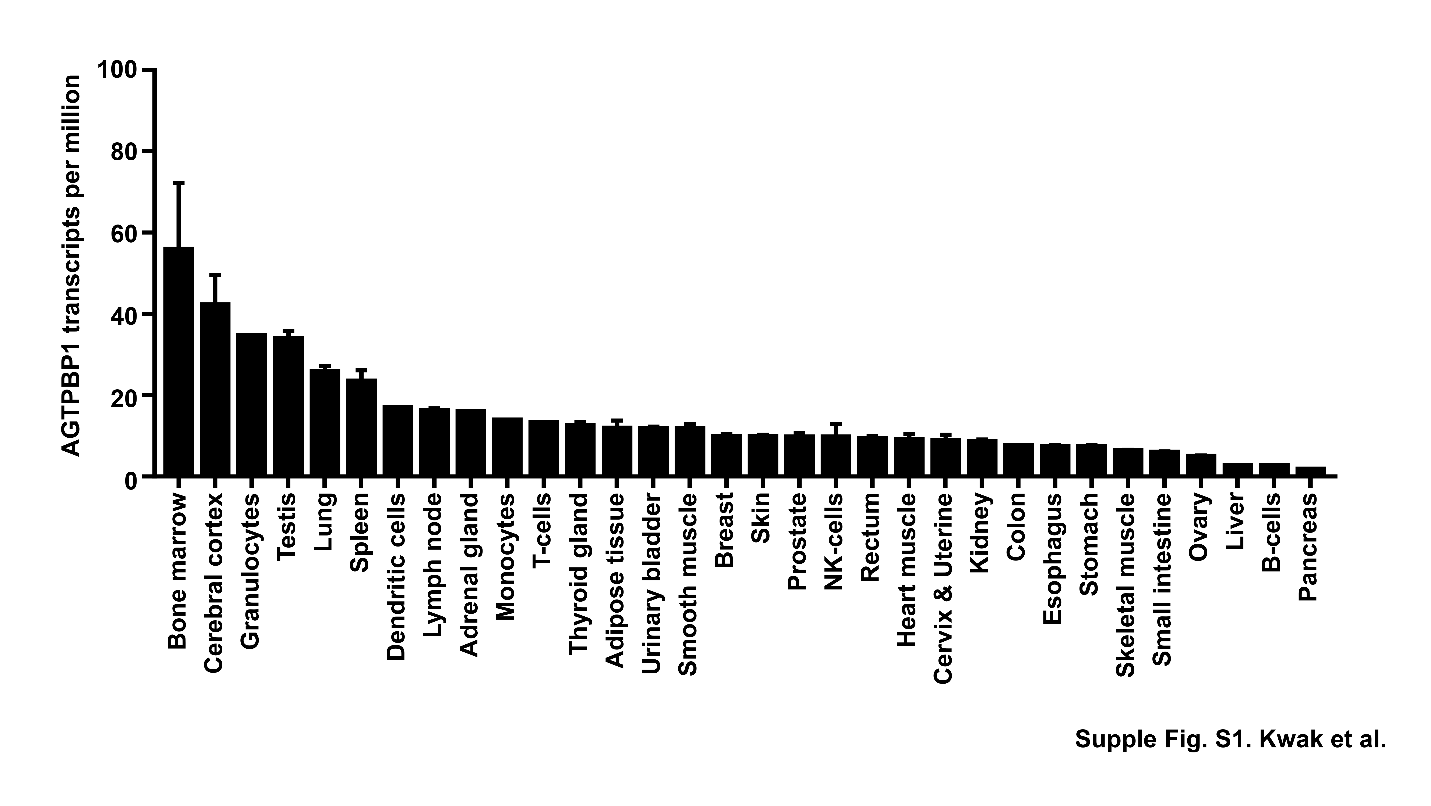
**

**Supplementary Figure S1.** Distribution of *AGTPBP1* expression in various normal tissue types through the HPA dataset. In total, 37 human tissues have been analyzed by RNA-seq to estimate the AGTPBP1 transcripts per million, corresponding to mean average values of the different individual samples from each tissue; Bone marrow, *n*=4; Cerebral Cortex, *n*=3; Testis, *n*=10; Lung, *n*=9; Spleen, *n*=5; Lymph node, *n*=5; Adrenal gland, *n*=1; Thyroid gland, *n*=5; Adipose tissue, *n*=5; Smooth muscle, *n*=3; Breast, *n*=4; Rectum, *n*=4; Heart Muscle, *n*=4; Cervix and Uterine, *n*=2; Kidney, *n*=9; Colon, *n*=13; Esophagus, *n*=3; Stomach, *n*=4; Skeletal muscle, *n*=5; Small intestine, *n*=4; Ovary, *n*=3; Liver, *n*=10; Pancreas, *n*=2.
