# Supplementary material for: Expression of ATP/GTP Binding Protein 1 Has Prognostic Value for the Clinical Outcomes in Non-Small Cell Lung Carcinoma
Source: J Pers Med. 2020 Dec 2;10(4):263. doi: 10.3390/jpm10040263 (PMC7761608; doi:10.3390/jpm10040263)
Supplement: Supplementary file 1 [file jpm-10-00263-s001.tgz › Supplementary Figure_S2.docx]

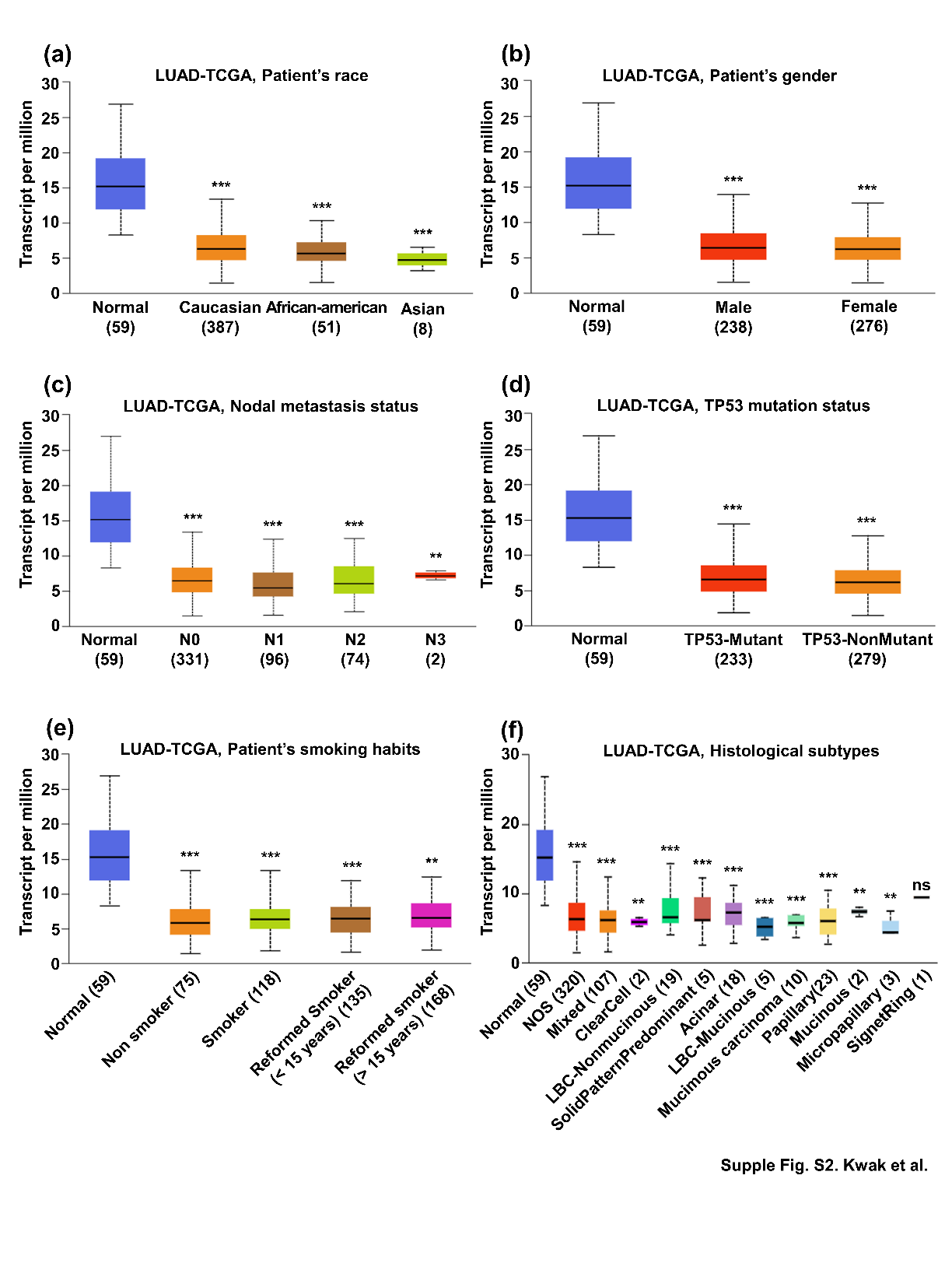


**Supplementary Figure S2**. The transcription level of *AGTPBP1* based on LUAD patient’s characteristics. The Box plot colors showed the normal tissues (blue) and clinicopathologic characteristics of LUAD (different color), such as (**a**) patient’s race, (**b**) patient’s gender, (**c**) nodal metastasis status, (**d**) TP53 mutation status, (**e**) patient’s smoking habits, and (**f**) histological subtypes. These data were acquired from TCGA using UALCAN web tool.
